# Solitary caecal diverticulitis as an unusual cause of a right iliac fossa mass: a case report

**DOI:** 10.1186/1752-1947-1-132

**Published:** 2007-11-10

**Authors:** Mohamed A Kurer

**Affiliations:** 1 Department of General Surgery, Colorectal Unit, York Hospital, York, YO31 8HE, UK

## Abstract

Inflammation of a solitary caecal diverticulum is an uncommon pathological condition. Preoperatively the condition is almost indistinguishable from appendicitis, and is often confused with carcinoma of the caecum during operation. The typical patient with this condition is male, Asian, and in the fourth decade of life. This case is unusual in that the patient was a 26-year-old Caucasian man.

## Case presentation

A 26-year-old white man was admitted to our hospital with a three day history of constant abdominal pain, which started centrally and shifted to the right lower quadrant. He had had recurrent episodes of abdominal pain for six months prior to his presentation. He had no vomiting or change of bowel habit, and no urinary symptoms. Physical examination revealed a temperature of 37.9°C, pulse 90 beats/min, and BP 130/80 mmHg. Marked right iliac fossa tenderness with rebound was the only significant physical finding. Digital rectal examination was normal. Apart from a raised WCC of 12.9 and CRP of 42, other initial investigations including urinalysis where within normal limits.

A presumptive diagnosis of appendicitis was made. Laparotomy revealed a right iliac fossa mass. There was a small amount of clear free fluid. The appendix was normal, as was the terminal ileum. There was a solitary diverticulum projecting from the medial aspect of the caecum and forming an inflammatory mass with the omentum. A limited right hemicolectomy was performed. The patient's recovery was uneventful. Examination of the resected specimen showed solitary caecal diverticulitis with perforation.

## Discussion

The first case of a solitary caecal diverticulum was reported in 1863; more than 500 cases have been recorded since then. The condition is uncommon, seen in only 10 of 5,000 radiological examinations, with greater incidence in Oriental people [1]. This condition is unrelated to the common and well known multiple colonic diverticular disease which is a common disorder in the West. According to experience in Singapore, the average age of presentation of caecal diverticulitis is 36 years (3–74) with male predominance [2]. Nearly 80% of caecal diverticula are situated in the area from one cm proximal to two cm distal to the ileocaecal valve [3]. They are usually solitary. Approximately 60% of solitary caecal diverticula arise from the anterior aspect of the caecum, so when inflamed they tend to perforate and cause peritonitis. However, a posteriorly situated caecal diverticulum may produce a mass without generalized peritonitis, simulating perforating carcinoma. Correct preoperative diagnosis of acute caecal diverticulitis is made only in about 9% of the patients, and most of these patients have had previous appendicectomy. Even peroperatively, the correct diagnosis is only made in about 65% of cases [4]. Although the aetiology of a solitary caecal diverticulum is uncertain, congenital origin is widely accepted. The theory is that an outgrowth of the caecum develops in the sixth week and atrophies by about the seventh week. If this 'transient appendix' fails to disappear, a diverticulum or a duplication of the appendix results [5-7]. This concept is supported by the fact that the condition has been reported in a 3-year-old girl [8], the incidence does not increase with age [9], and it is a true type of diverticulum. Most authors agree that, preoperatively, it is almost impossible to distinguish between acute solitary caecal diverticulitis and acute appendicitis, even retrospectively [10]. However, some authors believe that the longer duration of illness [11], the absence of nausea and vomiting [3], and the relative lack of toxicity [12], are features of caecal diverticulitis.

It has been suggested that chronic recurrent abdominal pain due to inflammation of a solitary caecal diverticulum might be responsible for the syndrome of "grumbling appendix". The outcome of chronic recurrent abdominal pain has been studied by many surgeons as well as gynaecologists often using laparoscopy. Caecal diverticula have not been encountered as a cause of the chronic recurrent abdominal pain. Adhesions, chronic and acute appendicitis, inguinal hernia, and endometriosis are the main causes of chronic recurrent abdominal pain [13].

There is no standard surgical procedure for the treatment of an inflamed solitary caecal diverticulum. The extent of the inflammation, the experience of the surgeon, and the confidence in the intraoperative diagnosis determine the procedure. Procedures range from simple diverticulectomy to right colectomy, with appendicectomy when only the diverticulum is removed [1]. Management of an incidentally found solitary caecal diverticulum is more controversial. The condition may be encountered preoperatively, e.g. following a barium enema examination, in which case it is probably sensible to do nothing. Whether to interfere or not, in the case of an asymptomatic solitary caecal diverticulum, should be based on the chances of it becoming inflamed or developing other complications. Although Canver and Freier claimed that 14% of caecal diverticula will ultimately become inflamed [14], they did not make it clear how they came to this conclusion. Perhaps the more important question is what we should do if a solitary caecal diverticulum is found incidentally at laparotomy. Langdon advised either excision or inversion [15]. However, because the problem is uncommon this approach is not evidence-based.

This case report illustrates the importance of being aware of solitary caecal diverticulitis as a differential diagnosis of a patient with a right iliac fossa mass presenting with acute abdominal pain. Simple diverticulectomy with appendicectomy may be adequate if the diagnosis is made with confidence. Right hemicolectomy is advisable if the diagnosis is uncertain or the inflammation is extensive.

## Conclusion

An acutely inflamed solitary caecal diverticulum is an uncommon cause of an acute abdomen in the western population. Clinically it is almost indistinguishable from acute appendicitis. The condition may present with a right iliac fossa mass and is likely to present a diagnostic challenge when encountered peroperatively, particularly to the trainee surgeons who are likely to be dealing with such cases. Although there are many treatment options, in the event of peroperative diagnostic uncertainty and in particular when neoplasia and granulomatous diseases can not be ruled out, a limited right hemicolectomy is advisable.

## Competing interests

The author(s) declare that they have no competing interests.

## Authors' contributions

MK managed the case and wrote the manuscript.
